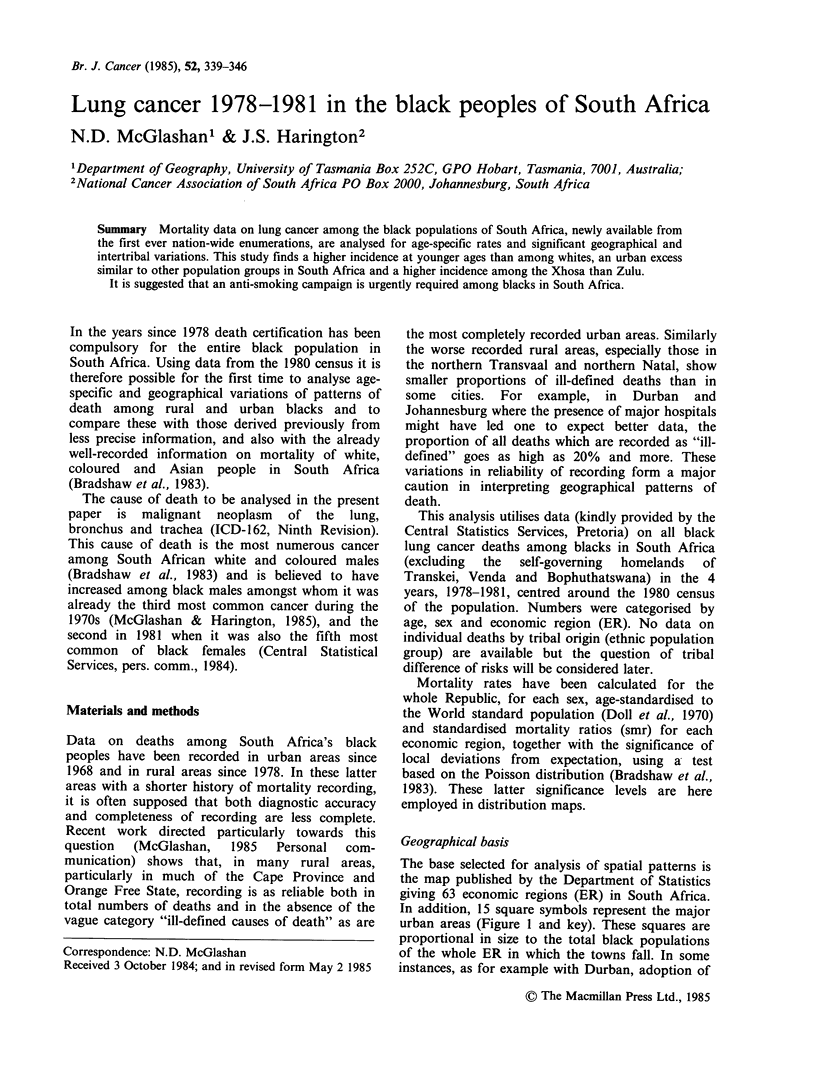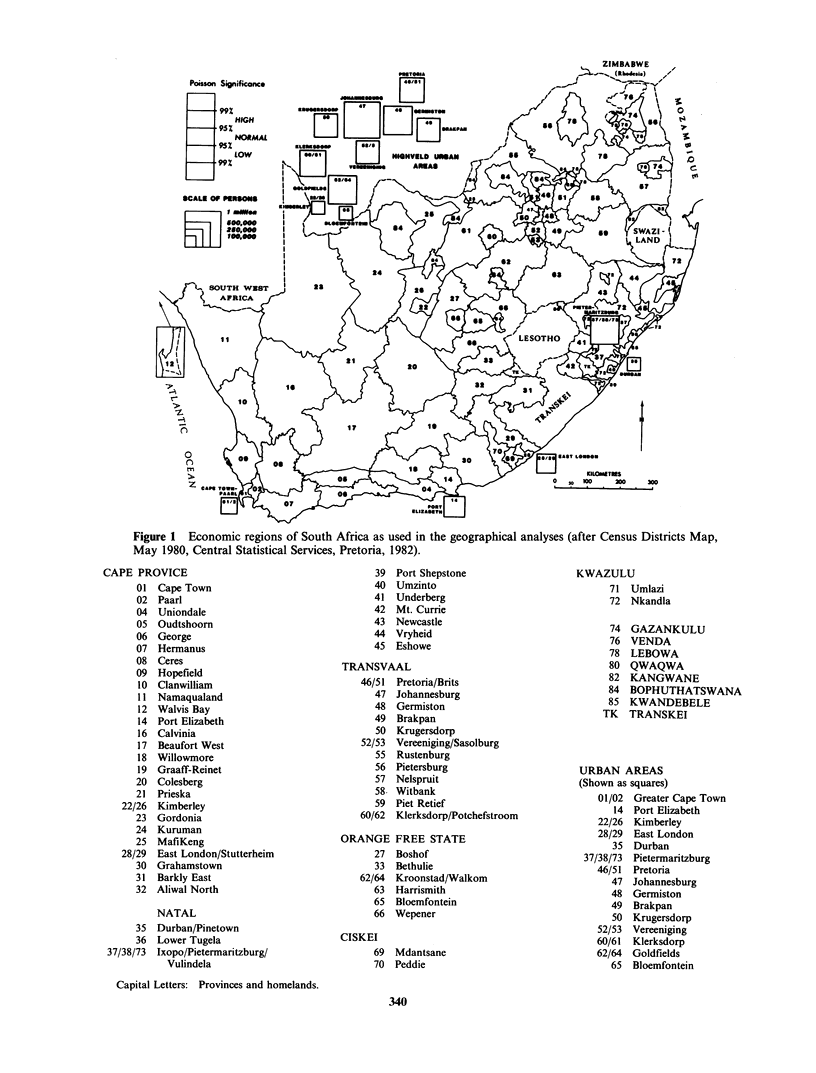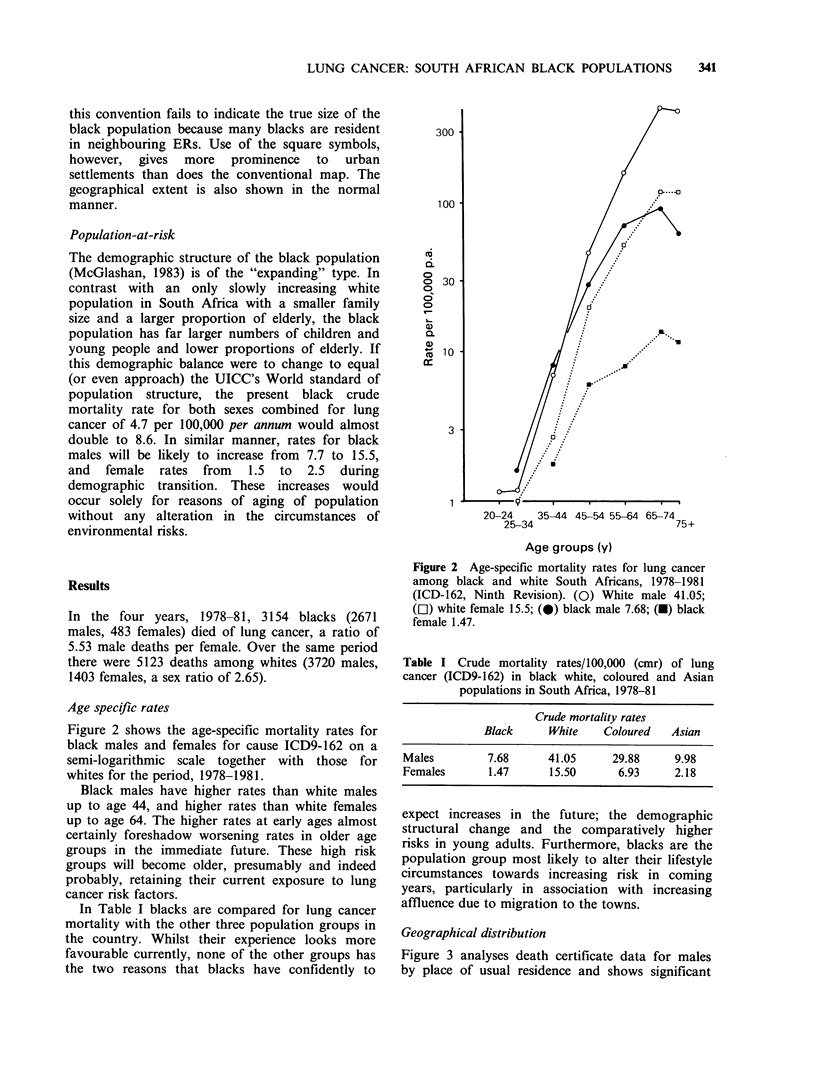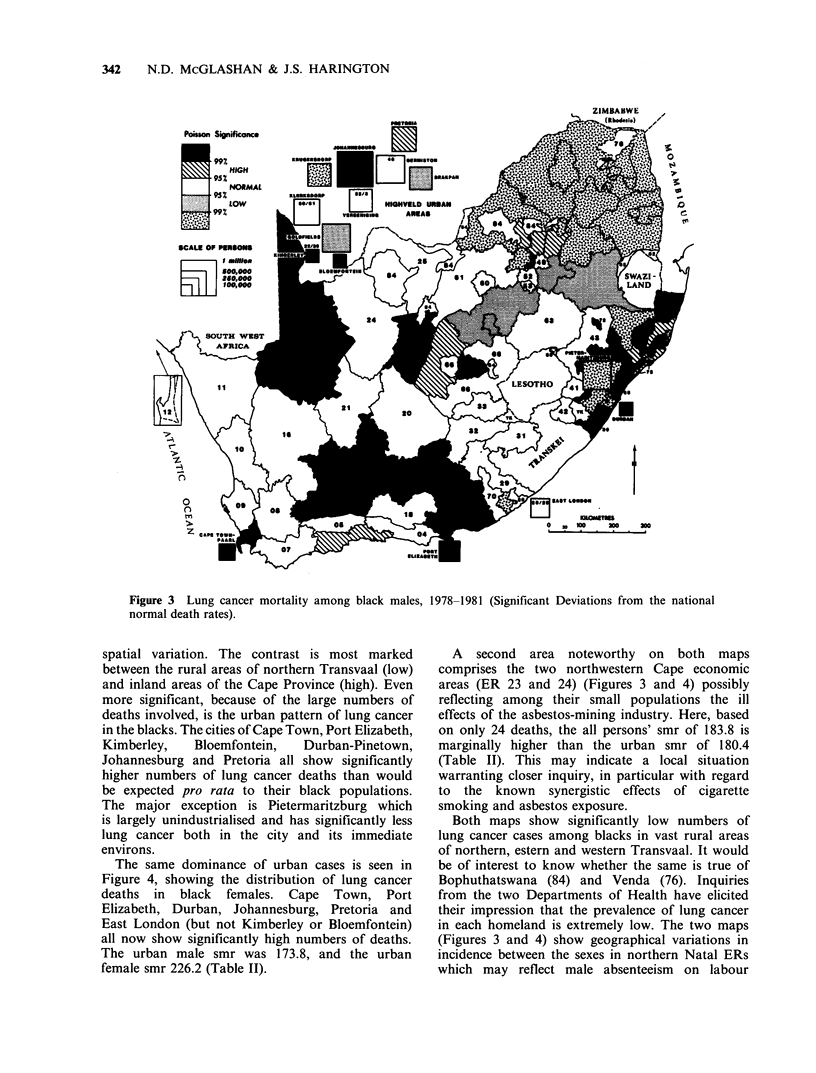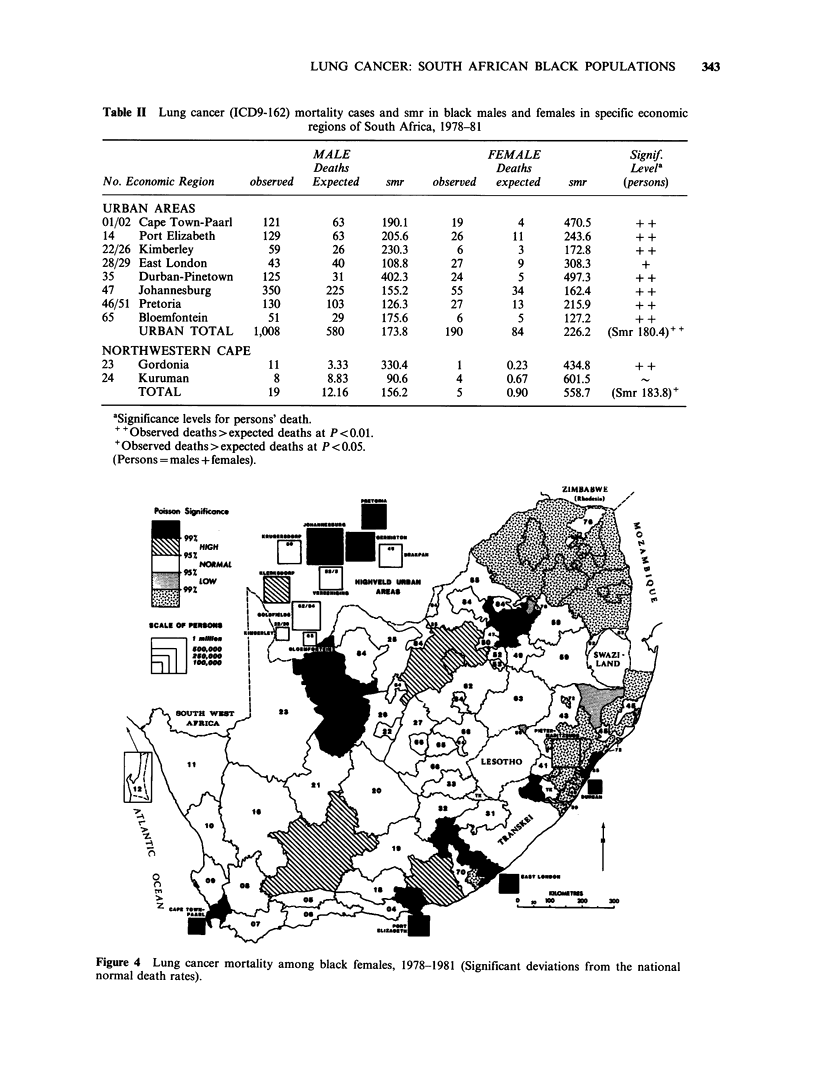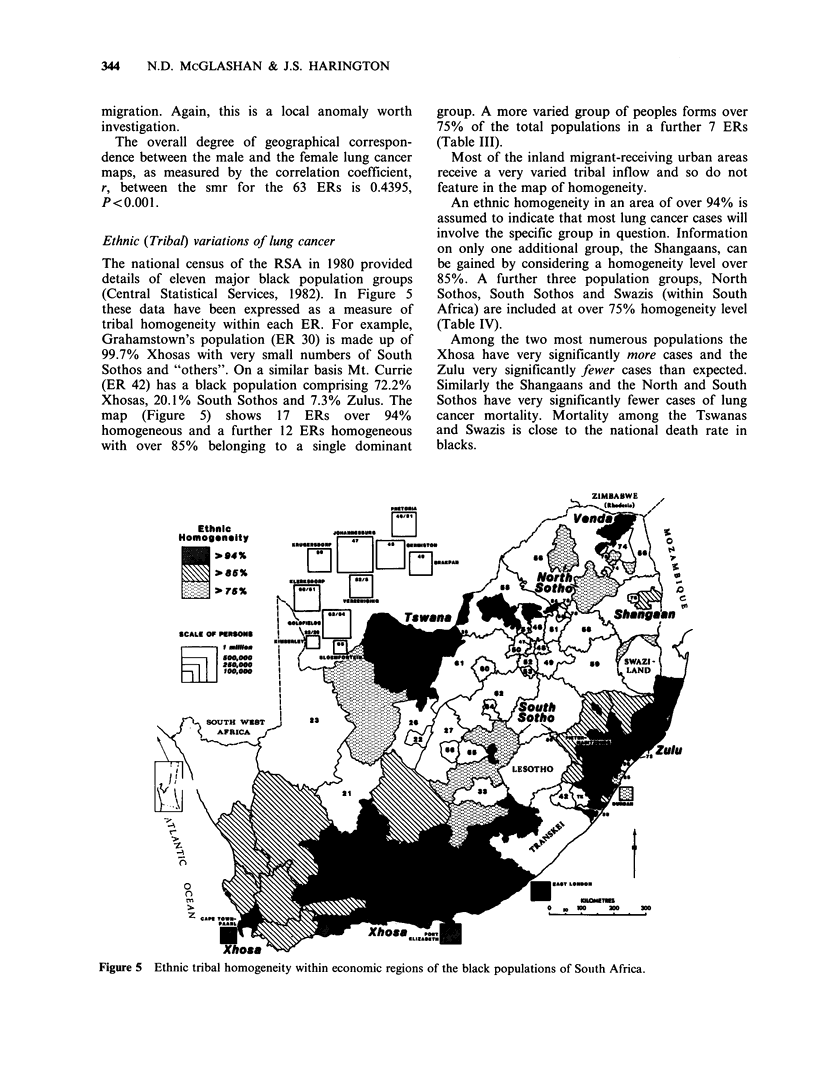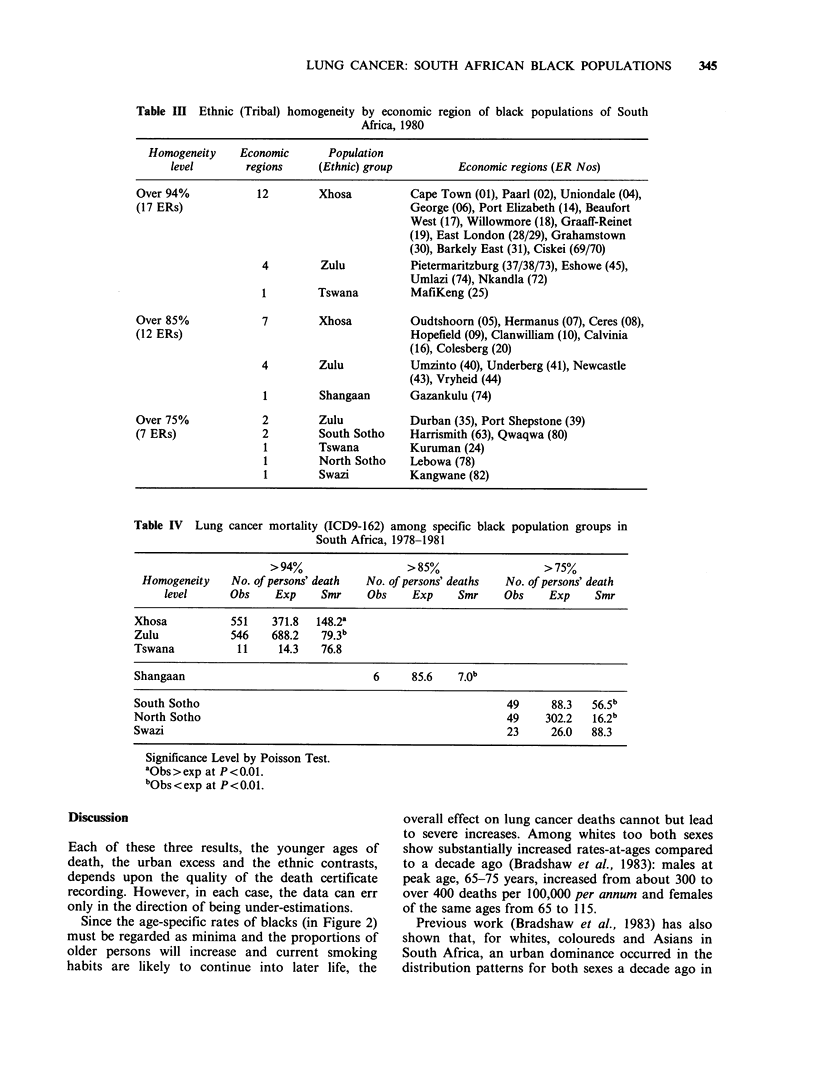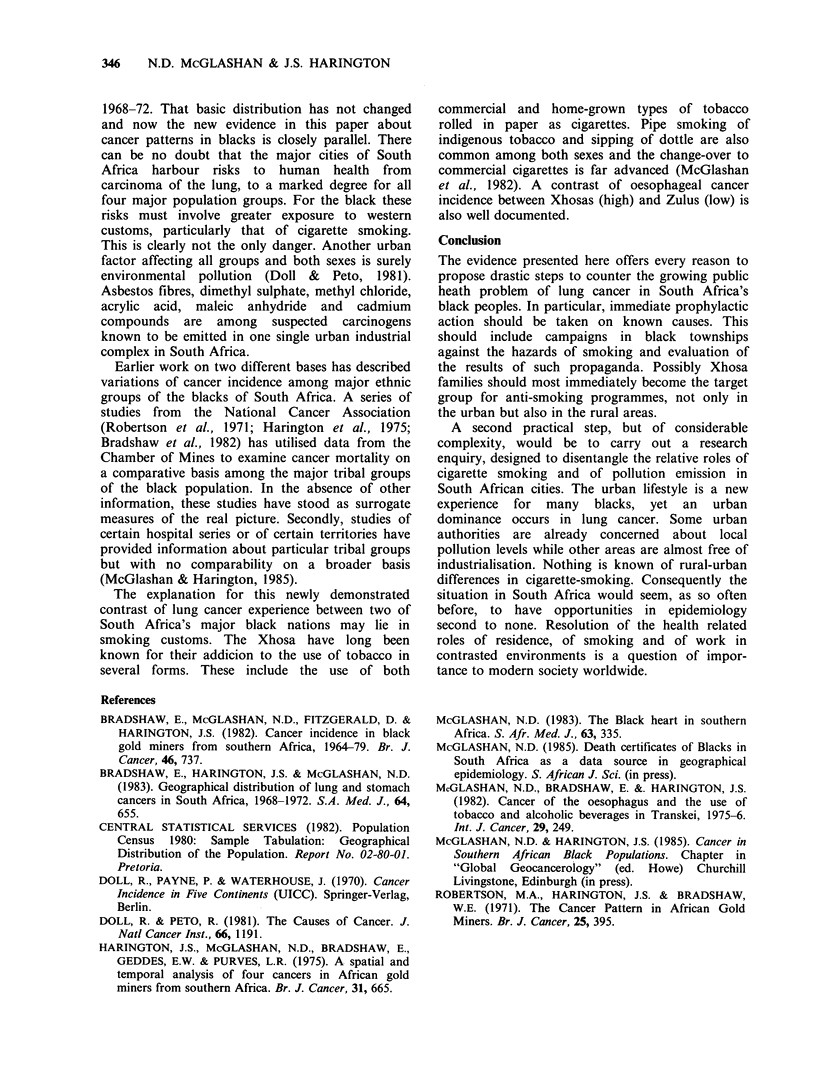# Lung cancer 1978-1981 in the black peoples of South Africa.

**DOI:** 10.1038/bjc.1985.199

**Published:** 1985-09

**Authors:** N. D. McGlashan, J. S. Harington

## Abstract

Mortality data on lung cancer among the black populations of South Africa, newly available from the first ever nation-wide enumerations, are analysed for age-specific rates and significant geographical and intertribal variations. This study finds a higher incidence at younger ages than among whites, an urban excess similar to other population groups in South Africa and a higher incidence among the Xhosa than Zulu. It is suggested that an anti-smoking campaign is urgently required among blacks in South Africa.


*Br. J. Cancer* (1985), **52**, 339–346

### Lung cancer 1978–1981 in the black peoples of South Africa

N.D. McGlashan[1] & J.S. Harington[2]

[1]*Department of Geography, University of Tasmania Box 252C, GPO Hobart, Tasmania, 7001, Australia;*
[2]*National Cancer Association of South Africa PO Box 2000, Johannesburg, South Africa*

**Summary** Mortality data on lung cancer among the black populations of South Africa, newly available from the first ever nation-wide enumerations, are analysed for age-specific rates and significant geographical and intertribal variations. This study finds a higher incidence at younger ages than among whites, an urban excess similar to other population groups in South Africa and a higher incidence among the Xhosa than Zulu.

It is suggested that an anti-smoking campaign is urgently required among blacks in South Africa.

In the years since 1978 death certification has been compulsory for the entire black population in South Africa. Using data from the 1980 census it is therefore possible for the first time to analyse age-specific and geographical variations of patterns of death among rural and urban blacks and to compare these with those derived previously from less precise information, and also with the already well-recorded information on mortality of white, coloured and Asian people in South Africa (Bradshaw *et al.*, 1983).

The cause of death to be analysed in the present paper is malignant neoplasm of the lung, bronchus and trachea (ICD-162, Ninth Revision). This cause of death is the most numerous cancer among South African white and coloured males (Bradshaw *et al.*, 1983) and is believed to have increased among black males amongst whom it was already the third most common cancer during the 1970s (McGlashan & Harington, 1985), and the second in 1981 when it was also the fifth most common of black females (Central Statistical Services, pers. comm., 1984).

### Materials and methods

Data on deaths among South Africa's black peoples have been recorded in urban areas since 1968 and in rural areas since 1978. In these latter areas with a shorter history of mortality recording, it is often supposed that both diagnostic accuracy and completeness of recording are less complete. Recent work directed particularly towards this question (McGlashan, 1985 Personal communication) shows that, in many rural areas, particularly in much of the Cape Province and Orange Free State, recording is as reliable both in total numbers of deaths and in the absence of the vague category "ill-defined causes of death" as are the most completely recorded urban areas. Similarly the worse recorded rural areas, especially those in the northern Transvaal and northern Natal, show smaller proportions of ill-defined deaths than in some cities. For example, in Durban and Johannesburg where the presence of major hospitals might have led one to expect better data, the proportion of all deaths which are recorded as "ill-defined" goes as high as 20% and more. These variations in reliability of recording form a major caution in interpreting geographical patterns of death.

This analysis utilises data (kindly provided by the Central Statistics Services, Pretoria) on all black lung cancer deaths among blacks in South Africa (excluding the self-governing homelands of Transkei, Venda and Bophuthatswana) in the 4 years, 1978–1981, centred around the 1980 census of the population. Numbers were categorised by age, sex and economic region (ER). No data on individual deaths by tribal origin (ethnic population group) are available but the question of tribal difference of risks will be considered later.

Mortality rates have been calculated for the whole Republic, for each sex, age-standardised to the World standard population (Doll *et al.*, 1970) and standardised mortality ratios (smr) for each economic region, together with the significance of local deviations from expectation, using a test based on the Poisson distribution (Bradshaw *et al.*, 1983). These latter significance levels are here employed in distribution maps.

### *Geographical basis*

The base selected for analysis of spatial patterns is the map published by the Department of Statistics giving 63 economic regions (ER) in South Africa. In addition, 15 square symbols represent the major urban areas (Figure 1 and key). These squares are proportional in size to the total black populations of the whole ER in which the towns fall. In some instances, as for example with Durban, adoption of

Correspondence: N.D. McGlashan
Received 3 October 1984; and in revised form May 2 1985

© The Macmillan Press Ltd., 1985

**Figure 1** Economic regions of South Africa as used in the geographical analyses (after Census Districts Map, May 1980, Central Statistical Services, Pretoria, 1982).

CAPE PROVICE

01 Cape Town
02 Paarl
04 Uniondale
05 Oudtshoorn
06 George
07 Hermanus
08 Ceres
09 Hopefield
10 Clanwilliam
11 Namaqualand
12 Walvis Bay
14 Port Elizabeth
16 Calvinia
17 Beaufort West
18 Willowmore
19 Graaff-Reinet
20 Colesberg
21 Prieska
22/26 Kimberley
23 Gordonia
24 Kuruman
25 MafiKeng
28/29 East London/Stutterheim
30 Grahamstown
31 Barkly East
32 Aliwal North

NATAL

35 Durban/Pinetown

36 Lower Tugela

37/38/73 Ixopo/Pietermaritzburg/ Vulindela

39 Port Shepstone

40 Umzinto

41 Underberg

42 Mt. Currie

43 Newcastle

44 Vryheid

45 Eshowe

TRANSVAAL

46/51 Pretoria/Brits
47 Johannesburg
48 Germiston
49 Brakpan
50 Krugersdorp
52/53 Vereeniging/Sasolburg
55 Rustenburg
56 Pietersburg
57 Nelspruit
58 Witbank
59 Piet Retief
60/62 Klerksdorp/Potchefstroom

ORANGE FREE STATE

27 Boshof
33 Bethulie
62/64 Kroonstad/Walkom
63 Harrismith
65 Bloemfontein
66 Wepener

CISKEI

69 Mdantsane
70 Peddie

KWAZULU

71 Umlazi
72 Nkandla

74 GAZANKULU

76 VENDA

78 LEBOWA

80 QWAQWA

82 KANGWANE

84 BOPHUTHATSWANA

85 KWANDEBELE

TK TRANSKEI

URBAN AREAS
(Shown as squares)

01/02 Greater Cape Town
14 Port Elizabeth
22/26 Kimberley
28/29 East London
35 Durban
37/38/73 Pietermaritzburg
46/51 Pretoria
47 Johannesburg
48 Germiston
49 Brakpan
50 Krugersdorp
52/53 Vereeniging
60/61 Klerksdorp
62/64 Goldfields
65 Bloemfontein

Capital Letters: Provinces and homelands.

this convention fails to indicate the true size of the black population because many blacks are resident in neighbouring ERs. Use of the square symbols, however, gives more prominence to urban settlements than does the conventional map. The geographical extent is also shown in the normal manner.

*Population-at-risk*

The demographic structure of the black population (McGlashan, 1983) is of the "expanding" type. In contrast with an only slowly increasing white population in South Africa with a smaller family size and a larger proportion of elderly, the black population has far larger numbers of children and young people and lower proportions of elderly. If this demographic balance were to change to equal (or even approach) the UICC's World standard of population structure, the present black crude mortality rate for both sexes combined for lung cancer of 4.7 per 100,000 *per annum* would almost double to 8.6. In similar manner, rates for black males will be likely to increase from 7.7 to 15.5, and female rates from 1.5 to 2.5 during demographic transition. These increases would occur solely for reasons of aging of population without any alteration in the circumstances of environmental risks.

### Results

In the four years, 1978–81, 3154 blacks (2671 males, 483 females) died of lung cancer, a ratio of 5.53 male deaths per female. Over the same period there were 5123 deaths among whites (3720 males, 1403 females, a sex ratio of 2.65).

*Age specific rates*

Figure 2 shows the age-specific mortality rates for black males and females for cause ICD9-162 on a semi-logarithmic scale together with those for whites for the period, 1978–1981.

Black males have higher rates than white males up to age 44, and higher rates than white females up to age 64. The higher rates at early ages almost certainly foreshadow worsening rates in older age groups in the immediate future. These high risk groups will become older, presumably and indeed probably, retaining their current exposure to lung cancer risk factors.

In Table I blacks are compared for lung cancer mortality with the other three population groups in the country. Whilst their experience looks more favourable currently, none of the other groups has the two reasons that blacks have confidently to expect increases in the future; the demographic structural change and the comparatively higher risks in young adults. Furthermore, blacks are the population group most likely to alter their lifestyle circumstances towards increasing risk in coming years, particularly in association with increasing affluence due to migration to the towns.

**Figure 2** Age-specific mortality rates for lung cancer among black and white South Africans, 1978–1981 (ICD-162, Ninth Revision). (○) White male 41.05; (□) white female 15.5; (●) black male 7.68; (■) black female 1.47.

**Table I** Crude mortality rates/100,000 (cmr) of lung cancer (ICD9-162) in black white, coloured and Asian populations in South Africa, 1978–81

| | *Crude mortality rates* | | | |
|---|---|---|---|---|
| | *Black* | *White* | *Coloured* | *Asian* |
| Males | 7.68 | 41.05 | 29.88 | 9.98 |
| Females | 1.47 | 15.50 | 6.93 | 2.18 |

*Geographical distribution*

Figure 3 analyses death certificate data for males by place of usual residence and shows significant

**Figure 3** Lung cancer mortality among black males, 1978–1981 (Significant Deviations from the national normal death rates).

spatial variation. The contrast is most marked between the rural areas of northern Transvaal (low) and inland areas of the Cape Province (high). Even more significant, because of the large numbers of deaths involved, is the urban pattern of lung cancer in the blacks. The cities of Cape Town, Port Elizabeth, Kimberley, Bloemfontein, Durban-Pinetown, Johannesburg and Pretoria all show significantly higher numbers of lung cancer deaths than would be expected *pro rata* to their black populations. The major exception is Pietermaritzburg which is largely unindustrialised and has significantly less lung cancer both in the city and its immediate environs.

The same dominance of urban cases is seen in Figure 4, showing the distribution of lung cancer deaths in black females. Cape Town, Port Elizabeth, Durban, Johannesburg, Pretoria and East London (but not Kimberley or Bloemfontein) all now show significantly high numbers of deaths. The urban male smr was 173.8, and the urban female smr 226.2 (Table II).

A second area noteworthy on both maps comprises the two northwestern Cape economic areas (ER 23 and 24) (Figures 3 and 4) possibly reflecting among their small populations the ill effects of the asbestos-mining industry. Here, based on only 24 deaths, the all persons' smr of 183.8 is marginally higher than the urban smr of 180.4 (Table II). This may indicate a local situation warranting closer inquiry, in particular with regard to the known synergistic effects of cigarette smoking and asbestos exposure.

Both maps show significantly low numbers of lung cancer cases among blacks in vast rural areas of northern, estern and western Transvaal. It would be of interest to know whether the same is true of Bophuthatswana (84) and Venda (76). Inquiries from the two Departments of Health have elicited their impression that the prevalence of lung cancer in each homeland is extremely low. The two maps (Figures 3 and 4) show geographical variations in incidence between the sexes in northern Natal ERs which may reflect male absenteeism on labour

**Table II** Lung cancer (ICD9-162) mortality cases and smr in black males and females in specific economic regions of South Africa, 1978–81

| *No.* | *Economic Region* | *MALE* *observed* | *MALE Deaths Expected* | *smr* | *FEMALE* *observed* | *FEMALE Deaths expected* | *smr* | *Signif. Level*[a] *(persons)* |
|---|---|---|---|---|---|---|---|---|
| URBAN AREAS | | | | | | | | |
| 01/02 | Cape Town-Paarl | 121 | 63 | 190.1 | 19 | 4 | 470.5 | + + |
| 14 | Port Elizabeth | 129 | 63 | 205.6 | 26 | 11 | 243.6 | + + |
| 22/26 | Kimberley | 59 | 26 | 230.3 | 6 | 3 | 172.8 | + + |
| 28/29 | East London | 43 | 40 | 108.8 | 27 | 9 | 308.3 | + |
| 35 | Durban-Pinetown | 125 | 31 | 402.3 | 24 | 5 | 497.3 | + + |
| 47 | Johannesburg | 350 | 225 | 155.2 | 55 | 34 | 162.4 | + + |
| 46/51 | Pretoria | 130 | 103 | 126.3 | 27 | 13 | 215.9 | + + |
| 65 | Bloemfontein | 51 | 29 | 175.6 | 6 | 5 | 127.2 | + + |
| | URBAN TOTAL | 1,008 | 580 | 173.8 | 190 | 84 | 226.2 | (Smr 180.4)[++] |
| NORTHWESTERN CAPE | | | | | | | | |
| 23 | Gordonia | 11 | 3.33 | 330.4 | 1 | 0.23 | 434.8 | + + |
| 24 | Kuruman | 8 | 8.83 | 90.6 | 4 | 0.67 | 601.5 | ~ |
| | TOTAL | 19 | 12.16 | 156.2 | 5 | 0.90 | 558.7 | (Smr 183.8)[+] |

[a]Significance levels for persons' death.
[++]Observed deaths > expected deaths at $P<0.01$.
[+]Observed deaths > expected deaths at $P<0.05$.
(Persons = males + females).

**Figure 4** Lung cancer mortality among black females, 1978–1981 (Significant deviations from the national normal death rates).

migration. Again, this is a local anomaly worth investigation.

The overall degree of geographical correspondence between the male and the female lung cancer maps, as measured by the correlation coefficient, *r*, between the smr for the 63 ERs is 0.4395, $P<0.001$.

### *Ethnic (Tribal) variations of lung cancer*

The national census of the RSA in 1980 provided details of eleven major black population groups (Central Statistical Services, 1982). In Figure 5 these data have been expressed as a measure of tribal homogeneity within each ER. For example, Grahamstown's population (ER 30) is made up of 99.7% Xhosas with very small numbers of South Sothos and "others". On a similar basis Mt. Currie (ER 42) has a black population comprising 72.2% Xhosas, 20.1% South Sothos and 7.3% Zulus. The map (Figure 5) shows 17 ERs over 94% homogeneous and a further 12 ERs homogeneous with over 85% belonging to a single dominant group. A more varied group of peoples forms over 75% of the total populations in a further 7 ERs (Table III).

Most of the inland migrant-receiving urban areas receive a very varied tribal inflow and so do not feature in the map of homogeneity.

An ethnic homogeneity in an area of over 94% is assumed to indicate that most lung cancer cases will involve the specific group in question. Information on only one additional group, the Shangaans, can be gained by considering a homogeneity level over 85%. A further three population groups, North Sothos, South Sothos and Swazis (within South Africa) are included at over 75% homogeneity level (Table IV).

Among the two most numerous populations the Xhosa have very significantly *more* cases and the Zulu very significantly *fewer* cases than expected. Similarly the Shangaans and the North and South Sothos have very significantly fewer cases of lung cancer mortality. Mortality among the Tswanas and Swazis is close to the national death rate in blacks.

**Figure 5** Ethnic tribal homogeneity within economic regions of the black populations of South Africa.

**Table III** Ethnic (Tribal) homogeneity by economic region of black populations of South Africa, 1980

| *Homogeneity level* | *Economic regions* | *Population (Ethnic) group* | *Economic regions (ER Nos)* |
|---|---|---|---|
| Over 94% (17 ERs) | 12 | Xhosa | Cape Town (01), Paarl (02), Uniondale (04), George (06), Port Elizabeth (14), Beaufort West (17), Willowmore (18), Graaff-Reinet (19), East London (28/29), Grahamstown (30), Barkely East (31), Ciskei (69/70) |
| | 4 | Zulu | Pietermaritzburg (37/38/73), Eshowe (45), Umlazi (74), Nkandla (72) |
| | 1 | Tswana | MafiKeng (25) |
| Over 85% (12 ERs) | 7 | Xhosa | Oudtshoorn (05), Hermanus (07), Ceres (08), Hopefield (09), Clanwilliam (10), Calvinia (16), Colesberg (20) |
| | 4 | Zulu | Umzinto (40), Underberg (41), Newcastle (43), Vryheid (44) |
| | 1 | Shangaan | Gazankulu (74) |
| Over 75% (7 ERs) | 2 | Zulu | Durban (35), Port Shepstone (39) |
| | 2 | South Sotho | Harrismith (63), Qwaqwa (80) |
| | 1 | Tswana | Kuruman (24) |
| | 1 | North Sotho | Lebowa (78) |
| | 1 | Swazi | Kangwane (82) |

**Table IV** Lung cancer mortality (ICD9-162) among specific black population groups in South Africa, 1978–1981

| *Homogeneity level* | >94% *No. of persons' death* *Obs* | *Exp* | *Smr* | >85% *No. of persons' deaths* *Obs* | *Exp* | *Smr* | >75% *No. of persons' death* *Obs* | *Exp* | *Smr* |
|---|---|---|---|---|---|---|---|---|---|
| Xhosa | 551 | 371.8 | 148.2[a] | | | | | | |
| Zulu | 546 | 688.2 | 79.3[b] | | | | | | |
| Tswana | 11 | 14.3 | 76.8 | | | | | | |
| Shangaan | | | | 6 | 85.6 | 7.0[b] | | | |
| South Sotho | | | | | | | 49 | 88.3 | 56.5[b] |
| North Sotho | | | | | | | 49 | 302.2 | 16.2[b] |
| Swazi | | | | | | | 23 | 26.0 | 88.3 |

Significance Level by Poisson Test.
[a]Obs > exp at $P<0.01$.
[b]Obs < exp at $P<0.01$.

### Discussion

Each of these three results, the younger ages of death, the urban excess and the ethnic contrasts, depends upon the quality of the death certificate recording. However, in each case, the data can err only in the direction of being under-estimations.

Since the age-specific rates of blacks (in Figure 2) must be regarded as minima and the proportions of older persons will increase and current smoking habits are likely to continue into later life, the overall effect on lung cancer deaths cannot but lead to severe increases. Among whites too both sexes show substantially increased rates-at-ages compared to a decade ago (Bradshaw *et al.*, 1983): males at peak age, 65–75 years, increased from about 300 to over 400 deaths per 100,000 *per annum* and females of the same ages from 65 to 115.

Previous work (Bradshaw *et al.*, 1983) has also shown that, for whites, coloureds and Asians in South Africa, an urban dominance occurred in the distribution patterns for both sexes a decade ago in

1968–72. That basic distribution has not changed and now the new evidence in this paper about cancer patterns in blacks is closely parallel. There can be no doubt that the major cities of South Africa harbour risks to human health from carcinoma of the lung, to a marked degree for all four major population groups. For the black these risks must involve greater exposure to western customs, particularly that of cigarette smoking. This is clearly not the only danger. Another urban factor affecting all groups and both sexes is surely environmental pollution (Doll & Peto, 1981). Asbestos fibres, dimethyl sulphate, methyl chloride, acrylic acid, maleic anhydride and cadmium compounds are among suspected carcinogens known to be emitted in one single urban industrial complex in South Africa.

Earlier work on two different bases has described variations of cancer incidence among major ethnic groups of the blacks of South Africa. A series of studies from the National Cancer Association (Robertson *et al.*, 1971; Harington *et al.*, 1975; Bradshaw *et al.*, 1982) has utilised data from the Chamber of Mines to examine cancer mortality on a comparative basis among the major tribal groups of the black population. In the absence of other information, these studies have stood as surrogate measures of the real picture. Secondly, studies of certain hospital series or of certain territories have provided information about particular tribal groups but with no comparability on a broader basis (McGlashan & Harington, 1985).

The explanation for this newly demonstrated contrast of lung cancer experience between two of South Africa's major black nations may lie in smoking customs. The Xhosa have long been known for their addicion to the use of tobacco in several forms. These include the use of both commercial and home-grown types of tobacco rolled in paper as cigarettes. Pipe smoking of indigenous tobacco and sipping of dottle are also common among both sexes and the change-over to commercial cigarettes is far advanced (McGlashan *et al.*, 1982). A contrast of oesophageal cancer incidence between Xhosas (high) and Zulus (low) is also well documented.

### Conclusion

The evidence presented here offers every reason to propose drastic steps to counter the growing public heath problem of lung cancer in South Africa's black peoples. In particular, immediate prophylactic action should be taken on known causes. This should include campaigns in black townships against the hazards of smoking and evaluation of the results of such propaganda. Possibly Xhosa families should most immediately become the target group for anti-smoking programmes, not only in the urban but also in the rural areas.

A second practical step, but of considerable complexity, would be to carry out a research enquiry, designed to disentangle the relative roles of cigarette smoking and of pollution emission in South African cities. The urban lifestyle is a new experience for many blacks, yet an urban dominance occurs in lung cancer. Some urban authorities are already concerned about local pollution levels while other areas are almost free of industrialisation. Nothing is known of rural-urban differences in cigarette-smoking. Consequently the situation in South Africa would seem, as so often before, to have opportunities in epidemiology second to none. Resolution of the health related roles of residence, of smoking and of work in contrasted environments is a question of importance to modern society worldwide.

### References

BRADSHAW, E., McGLASHAN, N.D., FITZGERALD, D. & HARINGTON, J.S. (1982). Cancer incidence in black gold miners from southern Africa, 1964–79. *Br. J. Cancer*, **46**, 737.

BRADSHAW, E., HARINGTON, J.S. & McGLASHAN, N.D. (1983). Geographical distribution of lung and stomach cancers in South Africa, 1968–1972. *S.A. Med. J.*, **64**, 655.

CENTRAL STATISTICAL SERVICES (1982). Population Census 1980: Sample Tabulation: Geographical Distribution of the Population. *Report No. 02-80-01. Pretoria.*

DOLL, R., PAYNE, P. & WATERHOUSE, J. (1970). *Cancer Incidence in Five Continents* (UICC). Springer-Verlag, Berlin.

DOLL, R. & PETO, R. (1981). The Causes of Cancer. *J. Natl Cancer Inst.*, **66**, 1191.

HARINGTON, J.S., McGLASHAN, N.D., BRADSHAW, E., GEDDES, E.W. & PURVES, L.R. (1975). A spatial and temporal analysis of four cancers in African gold miners from southern Africa. *Br. J. Cancer*, **31**, 665.

McGLASHAN, N.D. (1983). The Black heart in southern Africa. *S. Afr. Med. J.*, **63**, 335.

McGLASHAN, N.D. (1985). Death certificates of Blacks in South Africa as a data source in geographical epidemiology. *S. African J. Sci.* (in press).

McGLASHAN, N.D., BRADSHAW, E. &. HARINGTON, J.S. (1982). Cancer of the oesophagus and the use of tobacco and alcoholic beverages in Transkei, 1975–6. *Int. J. Cancer*, **29**, 249.

McGLASHAN, N.D. & HARINGTON, J.S. (1985). *Cancer in Southern African Black Populations*. Chapter in "Global Geocancerology" (ed. Howe) Churchill Livingstone, Edinburgh (in press).

ROBERTSON, M.A., HARINGTON, J.S. & BRADSHAW, W.E. (1971). The Cancer Pattern in African Gold Miners. *Br. J. Cancer*, **25**, 395.